# Matrix Completion Optimization for Localization in Wireless Sensor Networks for Intelligent IoT

**DOI:** 10.3390/s16050722

**Published:** 2016-05-18

**Authors:** Thu L. N. Nguyen, Yoan Shin

**Affiliations:** School of Electronic Engineering, Soongsil University, Seoul 156-743, Korea; thunguyen@ssu.ac.kr

**Keywords:** Internet of Things, wireless sensor network, localization, Euclidean distance matrix completion, semi-definite programming, modified Newton method

## Abstract

Localization in wireless sensor networks (WSNs) is one of the primary functions of the intelligent Internet of Things (IoT) that offers automatically discoverable services, while the localization accuracy is a key issue to evaluate the quality of those services. In this paper, we develop a framework to solve the Euclidean distance matrix completion problem, which is an important technical problem for distance-based localization in WSNs. The sensor network localization problem is described as a low-rank dimensional Euclidean distance completion problem with known nodes. The task is to find the sensor locations through recovery of missing entries of a squared distance matrix when the dimension of the data is small compared to the number of data points. We solve a relaxation optimization problem using a modification of Newton’s method, where the cost function depends on the squared distance matrix. The solution obtained in our scheme achieves a lower complexity and can perform better if we use it as an initial guess for an interactive local search of other higher precision localization scheme. Simulation results show the effectiveness of our approach.

## 1. Introduction

### 1.1. Localization in Wireless Sensor Networks

Localization of sensor nodes is a challenging issue in wireless sensor networks (WSNs) for intelligent Internet of Things (IoT). Localization systems are not only for location identification but also provide information for routing, density control, tracking and a number of other communication network applications which integrate in many technologies of IoT. In general, the sensor positioning process has two steps. First, we choose the signal parameters to describe the location information between sensors. Second, we use some parametric methods for estimating the sensor positions based on the signal parameters in the first step. For the first step, GPS-based localization systems have a high degree of accuracy and offer global location information. However, alternative solutions for GPS are required, which are cost effective, rapidly deployable and can operate in diverse conditions, especially for indoor or non-line-of-sight environments. For these reasons, more suitable localization algorithms for WSNs need to be investigated. Some existing localization approaches such as time-of-arrival (TOA), time-difference-of-arrival, and angle-of-arrival achieve accurate localization results, but require high cost, complicated timing and synchronization. On the other hand, received signal strength (RSS) measurements are quite simple to obtain [1,2,3].

In this paper, we study sensor network localization problems in embedding dimension, given anchors and the RSS information between sensors. The anchors are located at fixed known sensor positions, and distances between unknown sensors and anchors are estimated from RSS measurements. Our goal is to approximate all sensor locations by using only a partial Euclidean distance matrix for the second step. Recently, many solution techniques for this problem have been proposed, such as semidefinite programming relaxation (SDP) and solvers [4,5,6], multidimensional scaling (MDS) and its improvements [7,8,9], heuristics [10], Euclidean distance matrix completion (EDMC) [11], to name a few. However, most of previous approaches are not scalable and require high computational complexity, so that we try to reduce computational complexity by using a modified iterative Newton’s algorithm for a cost function.

*Notations*: The following notations are used throughout our paper. ${\left(\cdot \right)}^{T}$ and ${\left(\cdot \right)}^{-1}$ denote the transpose and the inverse operations. ∘ denotes the Hadamard product. $\left|\right|\cdot {\left|\right|}_{2}$ and $\left|\right|\cdot {\left|\right|}_{F}$ denote the $\ell_{2}$-norm and the Frobenius matrix norm, respectively. The notation $w∼N(\mu ,\sigma^{2})$ means that w is distributed according to the normal Gaussian distribution with the mean *μ* and the covariance $\sigma^{2}$. The operator diag(A) returns a column vector of the main diagonal elements of the matrix A. For vectors, argmin(x) returns the indices of the smallest elements in x. For two arbitrary symmetric matrices A and B, A⪰B means A-B is positive semidefinite. gradf and Hessf denote the gradient and the Hessian vectors representing the first and the second partial derivatives.

### 1.2. Euclidean Distance Matrix Completion

Consider a set of *n* points (or sensors) with locations $x_{1},\cdots ,x_{n}\in R^{r}$ (in practice, r=2 or 3). Denote $D\in R^{n\times n}$ as an Euclidean distance matrix whose entries are the squared pairwise distances between *n* points by setting

(1) $$D_{ij}=\left|\right|x_{i}-x_{j}{\left|\right|}_{2}^{2}$$

It is easy to see that the rank of D is upper bound by r+2 which is very small compared to the number of data points *n*, especially when *n* becomes large. The set of all Euclidean distance matrices in $R^{n\times n}$ is denoted as EDM(n). We associate a weighted undirected graph G=(V,E,W) with D, where the vertex set V={1,⋯,n}, the edge set E={(i,j):i≠j,andDisspecified}, and the positive edge weight $W=(w_{ij})$ with $w_{ij}=\sqrt{D_{ij}}$ for all (i,j)∈E. Let H be the matrix whose entries are

(2) $$h_{ij}=h_{ji}=\left\{\begin{array}{cc} 1\; & \text{if}\;(i,j)\in E\\ 0\; & \text{otherwise} \end{array}\right.$$

Given the observation data $\tilde{D}$, the low-rank distance matrix completion problem states as

(3) $$\min_{D\in \mathrm{EDM}(n)}||H\circ \left(\tilde{D}-D\right){\left|\right|}_{F}^{2}$$

To find out the relationship between positive semidefinite matrices and Euclidean distance matrices, let

(4) $$P=\left[\begin{array}{c} x_{1}^{T}\\ \vdots \\ x_{n}^{T} \end{array}\right]\;\text{and}\;Y=PP^{T}$$

Then, we can observe that

(5) $$D_{ij}=Y_{ii}-2Y_{ij}+Y_{jj}$$

Hence, D=κ(Y), where κ(·) is a linear map defined as

(6) $$\begin{array}{cc} \kappa :\;S^{n} & \to S^{n}\\ Y & \mapsto \kappa \left(Y\right)=\mathrm{diag}\left(Y\right)e^{T}+e\mathrm{diag}{\left(Y\right)}^{T}-2Y \end{array}$$

Here, $S^{n}=\left\{Y\in R^{n\times n}:Y=Y^{T}\right\}$ and $e\in R^{n}$ is the vector of all ones. The problem Equation (3) is equivalent to

(7) $$\min_{Y⪰0,Y\in E}||H\circ \left(\tilde{D}-\kappa \left(Y\right)\right){\left|\right|}_{F}^{2}$$

Since rank(D)≤r+2, we can solve a sequence of non-convex problems as the following rank constrained semidefinite optimization problem

(8) $$\begin{array}{cc} \min_{Y⪰0,Y\in E}\; & ||H\circ \left(\tilde{D}-\kappa \left(Y\right)\right){\left|\right|}_{F}^{2}\\ \text{subject}\;\text{to}\; & \mathrm{rank}(Y)=\rho \end{array}$$

By screening the value from ρ=1 to ρ=r+2, the solution presented in [12] guarantees a monotonic convergence to the original solution of Equation (7).

### 1.3. Contribution of the Paper

In order to solve Equation (7), several approaches have been studied in [4,5,6,7,8,9]. The conventional MDS method transforms the pairwise distance information into the relative coordinates of sensor nodes [4,5,6]. Thus, the global solution only obtained when we use all pairwise distance measurements of sensors, which is impractical. To overcome this problem, the authors in [7,8] proposed the distributed weighted MDS (WMDS/dwMDS) method, while the authors in [9] proposed the MDS-MAP. Both methods are basically based on either the linearized least square estimator for TOA information or the biased RSS-based estimators, so that the estimated solutions can be obtained explicit and much easier than the conventional MDS. However, their accuracy is not good when the variance of the measurement noise is large.

In this paper, our goal is to design a numerical estimator especially for the RSS-based localization systems. This estimator is based on the RSS measurements rather than pairwise distance information. We first formulate the sensor localization problem as a relaxation of SDP, then the solution can be numerically obtained via a modification of the Newton’s method as long as the RSS measurements are valid. This result is mainly used for coarse localization strategy, *i.e.*, reducing the region of interest and computation time for the fine localization stage, where the estimated solutions can be used as a starting point for other fine localization algorithms such as dwMDS and MDS-MAP, *etc*. This localization scheme provides a low-cost, low-complexity, and easy-implementation solution, thus there also exists tradeoff between the location accuracy and the computation complexity compared with other techniques.

The organization of this paper is as follows. In Section 2, we formulate the sensor network localization problem as a reduction of SDP. In Section 3, we describe our approach for node localization in WSNs by using a modification of the Newton’s method. Then, numerical evaluation results are presented in Section 4, followed by concluding remarks in Section 5.

## 2. Problem Formulation of Sensor Network Localization

Consider a sensor network consisting of *n* wireless sensors at the locations $p_{1},\cdots ,p_{n}\in R^{r}$, where *r* is the embedding dimension. As defined in the previous section, D represents the Euclidean distances between sensors within given radio range *R*. The embedding dimension is the smallest integer *r* satisfying

$$r=\min\{t:\exists p_{1},\cdots ,p_{n}\in R^{r}:D_{ij}=||p_{i}-p_{j}|{|}_{2}^{2},\;\forall i,j\}$$

We denote the locations of *m* known anchor nodes as $a_{1},\cdots ,a_{m}\in R^{r}$. Let $A^{T}=\left[a_{1},\cdots ,a_{m}\right]$, and $X^{T}=\left[p_{1},\cdots ,p_{n-m}\right]$ represents the unknown sensor nodes. In the sensor network localization problems, we ignore the distinction between the anchors and the other sensors, hence we identify $a_{i}$ with $p_{n-m+i}\;\left(i=1,\cdots ,m\right)$ and set

(9) $$P^{T}=\left[X^{T}\;A^{T}\right]\in R^{n\times r}$$

We also assume that there are a sufficient number of anchors, which makes our problem not be realized in a smaller embedding dimension.

Denoting the distance between the unknown node *i* and the anchor *j* as $\tau_{ij}$, the corresponding RSS measurement can be expressed according to the following radio propagation path loss model in dB’s [13].

(10) $$L_{ij}=L_{0}+10\gamma \log_{10}\frac{\tau_{ij}}{\tau_{0}}+\nu_{ij}$$

where $L_{ij}=P_{T}-P_{ij}$ is the path loss, $P_{T}$ is the transmission power, $L_{0}$ denotes the path loss value at the reference distance $\tau_{0}$, *γ* is the path loss exponent, and $\nu_{ij}$ is a Gaussian random variable representing the log-normal shadowing effect, $\nu_{ij}∼N\left(0,\sigma_{ij}^{2}\right)$. The distance $\tau_{ij}$ can be computed through the RSS measurement by the maximum likelihood (ML) estimation. Then, the corresponding ML estimator ${\hat{\tau }}_{ij}$ is given by [5]

(11) $$\begin{array}{cc} {\tilde{\tau }}_{ij} & =\arg\min_{\tau_{ij}}{\left[10\gamma \log_{10}\frac{\tau_{ij}}{\tau_{0}}-\left(L_{ij}-L_{0}\right)\right]}^{2} \end{array}$$

(12) $$\begin{array}{cc} & =\tau_{0}10^{(L_{ij}-L_{0})/10\gamma } \end{array}$$

Note that if we define $\xi_{ij}=\exp\left\{\frac{\nu_{ij}}{10\log_{10}e}\right\}$, where *e* is the Euler’s number, then $10\log_{10}\xi ∼N\left(0,\sigma_{ij}^{2}\right)$. The Equation (12) can be rewritten as

(13) $${\tilde{\tau }}_{ij}=\tau_{ij}\xi_{ij}^{1/\gamma }=\tau_{ij}{\left(\exp\left\{\frac{\nu_{ij}}{10\log_{10}e}\right\}\right)}^{1/\gamma }$$

By using the Gaussian moment generating function, we can derive

(14) $$E\left({\tilde{\tau }}_{ij}\right)=\tau_{ij}\exp\left\{\frac{\sigma_{ij}^{2}}{10\gamma^{2}\log_{10}^{2}e}\right\},\mathrm{Var}\left({\tilde{\tau }}_{ij}\right)=\tau_{ij}^{2}\left[\exp\left\{\frac{\sigma_{ij}^{2}}{50\gamma^{2}\log_{10}^{2}e}\right\}-\exp\left\{\frac{\sigma_{ij}^{2}}{100\gamma^{2}\log_{10}^{2}e}\right\}\right]$$

which shows that the estimator is biased. In order to obtain the unbiased estimator, we let ${\hat{\tau }}_{ij}=\frac{{\tilde{\tau }}_{ij}}{\varpi }$ where $\varpi =\exp\left\{\frac{\sigma_{ij}^{2}}{200\gamma^{2}\log_{10}^{2}e}\right\}$, which yields the following variance of $\mathrm{Var}\left({\hat{\tau }}_{ij}\right)=\tau_{ij}^{2}\left[\exp\left\{\frac{\sigma_{ij}^{2}}{100\gamma^{2}\log_{10}^{2}e}\right\}-1\right]$.

After obtaining the estimated distance ${\hat{\tau }}_{ij}$, we can apply the SDP estimator [6] or its relaxation [5,14] to reconstruct the distance matrix D from the RSS measurements. By partitioning, the n×n Euclidean squared distance matrix can be expressed as follows.

(15) $$D=\left[\begin{array}{cc} D_{11} & D_{12}\\ D_{21} & D_{22} \end{array}\right]$$

where $D_{11}$ is the (n-m)×(n-m) distance sub-matrix between the unknown locations, $D_{21}=D_{12}^{T}$ is the distance sub-matrix between the anchor locations and the unknown locations, and $D_{22}$ is the distance sub-matrix between the anchor locations.

However, since at any time an unknown node *i* is only in the communication range of small subset of the anchor nodes, the matrix of RSS measurements is partially known. Thus, the matrix D in Equation (8) is incomplete and is affected by noises, *i.e.*, $\tilde{D}=D+N$. Our goal is to reconstruct the complete distance matrix D from the RSS measurements. From this matrix, we can recover the unknown node locations. The problem formulation is briefly described as follows. Given $A\in R^{m\times r}$ and n×n distance matrix $\tilde{D}$ with corresponding adjacency matrix H, we need to solve

(16) $$\min_{D\in \mathrm{EDM}(n)}||H\circ \left(\tilde{D}-D\right){\left|\right|}_{F}^{2}$$

## 3. Main Results

The problem in Equation (16) cannot be solved easily due to its non-convex nature and high computational complexity. Then, we try to reformulate it into another form without leaving the minimum. We suppose that $P\in R^{n\times r}$ satisfies Pe=0 (centroid) and $H\circ {\left(PP\right)}^{T}=H\circ D$. Based on the fact that any rank-*r* positive semidefinite matrix admits a factorization $Y=PP^{T}$ [11], we rewrite Equation (16) as

(17) $$\begin{array}{cc} & \min_{D\in \mathrm{EDM}(n)}\;||H\circ \left(\tilde{D}-\kappa \left(PP^{T}\right)\right){\left|\right|}_{F}^{2}\\ & \text{subject}\;\text{to}\;P=\left[\begin{array}{c} X\\ A \end{array}\right]\in R^{n\times r},\mathrm{Pe}=0 \end{array}$$

For simplicity, let $p_{i}=\left[p_{i}^{\left(1\right)},\cdots ,p_{i}^{\left(r\right)}\right],D=\left[d_{ij}\right],H=\left[h_{ij}\right],\tilde{D}=\left[{\tilde{d}}_{ij}\right]$, and $Y=[y_{ij}]$. Therefore, we denote the objective function as

(18) $$\begin{array}{cc} f(P) & =||H\circ \left(\tilde{D}-\kappa \left(PP^{T}\right)\right){\left|\right|}_{F}^{2}\\ & =\sum_{i=1}^{n}\sum_{j=1}^{n}h_{ij}{\left({\tilde{d}}_{ij}-d_{ij}\right)}^{2}\\ & =\sum_{i=1}^{n}\sum_{j=1}^{n}h_{ij}({\tilde{d}}_{ij}-\left|\right|p_{i}-p_{j}{\left|\right|}^{2}{)}^{2} \end{array}$$

Following Equation (5), we have

(19) $$\begin{array}{cc} d_{ij} & =y_{ii}-2y_{ij}+y_{jj}\\ & =\sum_{k=1}^{r}{\left(p_{i}^{\left(k\right)}\right)}^{2}+\sum_{k=1}^{r}{\left(p_{j}^{\left(k\right)}\right)}^{2}-2\sum_{k=1}^{r}p_{i}^{\left(k\right)}p_{j}^{\left(k\right)} \end{array}$$

where i,j=1,⋯,n. This problem is now reformulated as an constrained optimization for the cost function Equation (18), which can be solved efficiently by using the Newton’s method [15,16,17]. One of the motivations behind this method for optimization is to describe it as a sequence of second-order Taylor expansions and minimization.

(20) $$f\left(P\right)\approx f\left(P_{k}\right)+\mathrm{grad}f{\left(P^{\left(k\right)}\right)}^{T}\xi +\frac{1}{2}\xi^{T}\mathrm{Hess}f\left(P_{k}\right)\xi$$

where $\xi =P-P_{k}$. The iteration that computes an approximate solution to the system of equations gradf(P)=0, is updated as

(21) $$P_{k+1}=P_{k}-{\left[\mathrm{Hess}f\left(P_{k}\right)\right]}^{-1}\mathrm{grad}f\left(P_{k}\right)$$

In practice, one does not compute $P_{k+1}$ by explicitly computing ${\left[\mathrm{Hess}f\left(P_{k}\right)\right]}^{-1}$ and then multiplying by $\mathrm{grad}f(P_{k})$, since it is computationally inefficient. Instead, it is more practical to solve the system of linear equations

(22) $$\mathrm{Hess}f\left(P_{k}\right)\xi_{k}=-\mathrm{grad}\left(P_{k}\right)$$

for unknown $\xi_{k}$. There are also many approaches such as matrix decompositions or other algebra techniques [18] in calculating ${\left[\mathrm{Hess}f\left(P_{k}\right)\right]}^{-1}$ to reduce its complexity when computing matrix operators. In general, the Newton’s method will find the minimum in one iteration and has extremely fast convergence, if Hessf(·) is positive definite at the minimum and the initial guess for P is close enough to this point. However, in some cases it may return any critical points of *f*, that is, minima, maxima, or saddle points. To avoid these situations, we make some modifications to the Newton’s method, so that it finds the critical points which are actually the minima.

For search direction, we perform a line search, *i.e.*, for each iteration we solve Equation (22) and update

(23) $$P_{k+1}=P_{k}+t\xi_{k}$$

where *t* is small enough to get fast convergence. It can be $1,\frac{1}{2}$ or $2^{-j},j=0,1,2,\cdots$ for some special cases [19].

It is also necessary to find an effective method to solve Equation (22). The Cholesky factorization is one of standard effective methods for solving linear systems compared to the Gaussian elimination or the LU decomposition. Note that any symmetric and positive definite matrix A can be expressed as $A=L\Sigma L^{T}$ for some unit lower triangular matrix L and diagonal matrix **Σ** with positive entries on the diagonal. For each step, we decompose $\mathrm{Hess}f(P_{k})$ as $\mathrm{Hess}f\left(P_{k}\right)=L_{k}\Sigma_{k}L_{k}^{T}$, then solve the following three sub-equations.

(24) $$\begin{array}{cc} & L_{k}\xi_{k}^{{}^{\prime }}=-\mathrm{grad}f\left(P_{k}\right) \end{array}$$

(25) $$\begin{array}{cc} & \Sigma_{k}\xi_{k}^{{}^{\prime \prime }}=\xi_{k}^{{}^{\prime }} \end{array}$$

(26) $$\begin{array}{cc} & L_{k}\xi_{k}=\xi_{k}^{{}^{\prime \prime }} \end{array}$$

so that $\Sigma_{k}L_{k}^{T}\xi_{k}=\xi_{k}^{{}^{\prime }}$, and $L_{k}^{T}\xi_{k}=\xi_{k}^{{}^{\prime \prime }}$. However, when $\mathrm{Hess}f(P_{k})$ is not positive definite, a Cholesky decomposition cannot be performed since its eigenvalues are sometimes very small negative numbers caused by rounding in computing and noise in the data. To overcome this kind of problems, we define a positive integer number *p* to avoid the computation error by replacing $\Sigma_{k}$ by *p* when $\Sigma_{k}$ is non-positive definite, and continue the factorization. If the absolute value of $\mathrm{grad}f(P_{k})$ is small or $P_{k+1}$ is close to $P_{k}$, then we terminate $P_{k+1}$ as the minimum, otherwise we continue the iteration. We always wish to construct $P_{k}$ with the convergence to the minimum at a rapid rate, so that few iterations are needed until the stopping criterion is satisfied. However, this has to be counterbalanced with the computation cost per iteration because of the trade-off between fast convergence and higher computational cost per iteration.

In Equation (17), we can discard the constraint Pe=0 during the computational process. The reason is explained as follows. Let us assume that $P\in R^{n\times r}$ is partitioned as

$$P=\left[\begin{array}{c} P_{1}\\ P_{2} \end{array}\right]$$

where $P_{1}\in R^{(n-m)\times r}$ and $P_{2}\in R^{m\times r}$. Since $H\circ {\left(PP\right)}^{T}=H\circ D$, we have $\kappa \left(P_{2}P_{2}^{T}\right)=\kappa \left(AA^{T}\right)$. If $A^{T}e=P_{2}e=0$, it imples that $P_{2}P_{2}^{T}=AA^{T}$, *i.e.*, there exists an orthogonal transformation Q satisfying $P_{2}Q=A$. It was well-known as the ProCrustes problem [4]. Hence, the problem Equation (17) becomes an unconstrained optimization and it can be solve efficiently by the modified Newton’s method. However, the equality constraint Pe=0 reduces the flexibility of P and can help the convergence to the global minimum in some cases.

**Figure 1:**
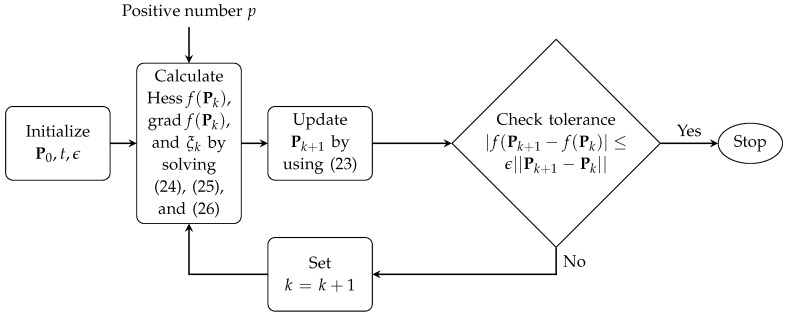
Procedures to solve the localization problem Equation (17).

In summary, we use the following procedures to solve the localization problem Equation (17) as illustrated in Figure 1.

**Remark 1.**  The modification of the Newton’s method is illustrated by the solution path on the function in [19], which is able to follow the shape of the U-valley and converges to the minimum using only finite difference gradients. For the deployment of other adaptive methods for the mesh refinement or the discrete Newton-like method, we will consider these methods as well as their convergence issues in the future work.

**Remark 2.**  It has been shown that the conventional MDS has the time complexity of $O(n^{3})$, while the WMDS [7] performed well for sparse networks but it is about two orders of magnitude slower than the MDS for larger networks (more than 100 node-networks). This is because the refinement strategy in [7] leads to a tradeoff between solution quality and computation cost. For the dwMDS algorithm [8], the authors proved that it takes $O(nL+n^{2}Ld_{thr}e_{dwMDS})$ time, where L is the number of iterations required until the stopping rule is satisfied, $d_{thr}=O\left(n^{1/r}\right)$ is the threshold distance, and $e_{dwMDS}$ depends on $d_{thr}$ in a nonlinear way. A drawback of the dwMDS is that complexity, convergence time and initial estimate requirements for each transmission time depend sensitively on heavy weights and range measurement accuracy. For our approach, it can be verified that the computing for matrix decomposition takes $O(n^{\kappa })$, where 1≤κ<3, thus the total cost can be computed as $O(Kn^{\kappa })$, where K is the number of iterations. Thus, our approach compares favorably to the conventional methods in terms of the complexity.

## 4. Numerical Evaluation

**Figure 2:**
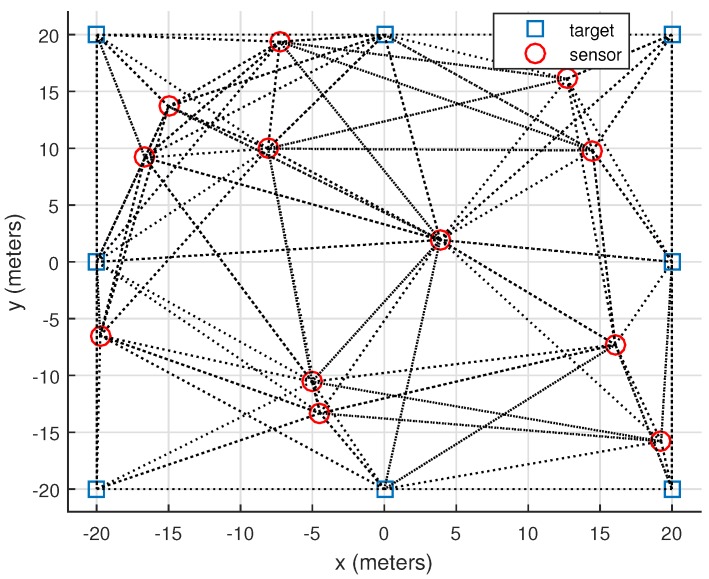
Geometry of a wireless sensor network for numerical evaluation.

In this section, the effectiveness of the proposed localization approach based on the modified Newton’s method is numerically investigated. Moreover, its location estimator is employed and compared. We consider a WSN consisting of 20 nodes placed in an area [-20,20]×[-20,20] m${}^{2}$ with 8 of them are anchors and its geometry is illustrated in Figure 2, *i.e.*, n=20,m=8 and n-m=12. The anchor locations are (20,20), (−20, −20), (20, 0), (−20, 0), (20, −20), (20, −20), (0, 20), (0, −20). The unknown sensor locations are randomly placed in this area. The maximum communication range between the nodes is set to be 25 meters. For the parameters in the path loss model of Equation (10), $L_{0}=-36.029$ dB, $\gamma =2.386,d_{0}=1$ meter and $\sigma_{ij}=\sigma$ for i,j=1,⋯,n. We define the root mean square error (RMSE) by

(27) $$\mathrm{RMSE}={\left(\frac{1}{n-m}\sum_{i=1}^{n-m}\left|\right|p_{i}^{\text{actual}}-p_{i}^{\text{estimated}}{\left|\right|}_{2}^{2}\right)}^{\frac{1}{2}}$$

**Figure 3:**
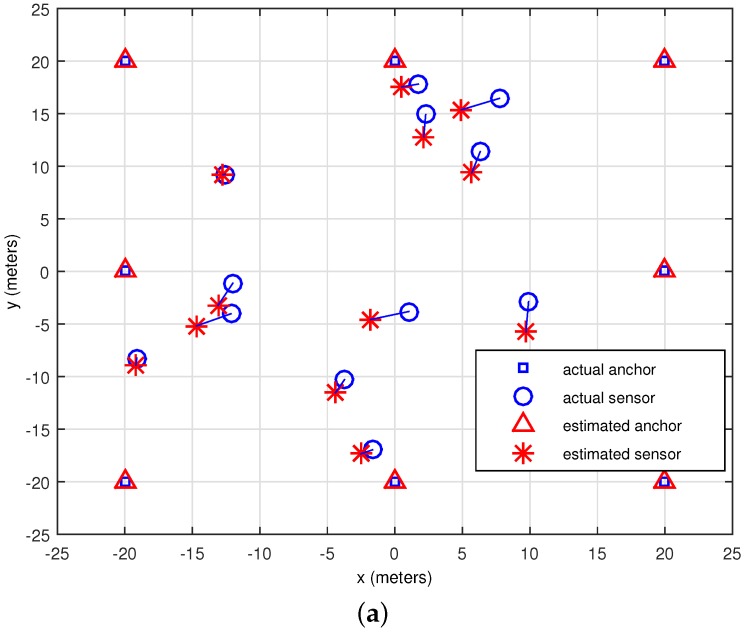

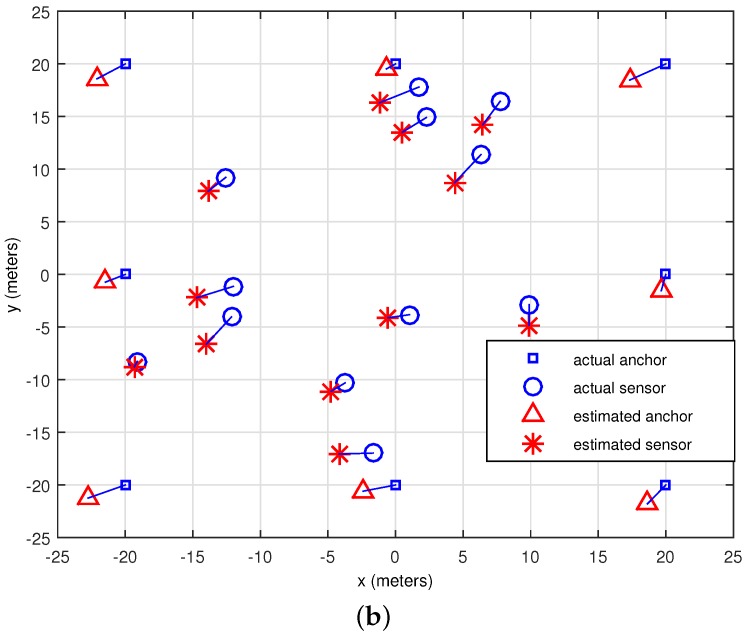
Single trial localization performance of the proposed scheme with σ=3.98 dB. (**a**) Presence of anchor locations (See text for details); (**b**) Absence of anchor locations (See text for details).

In the first experiment, we investigate the performance of the proposed algorithm as shown in Figure 3. It shows the effectiveness of the location estimation of our proposed scheme for a single trial with σ=3.98 dB [13] in two cases: (a) presence of anchor locations and (b) absence of anchor locations. By “presence of anchor locations”, we mean that these locations are used in the initial guess for P, and inverse for “absence of anchor locations” case. A maximum RMSEs of 1.4854 meter and 1.8833 meter are observed over 100 simulations in Figure 3a,b, respectively. It indicates that better initial points leads to better final results for our proposed scheme.

**Figure 4:**
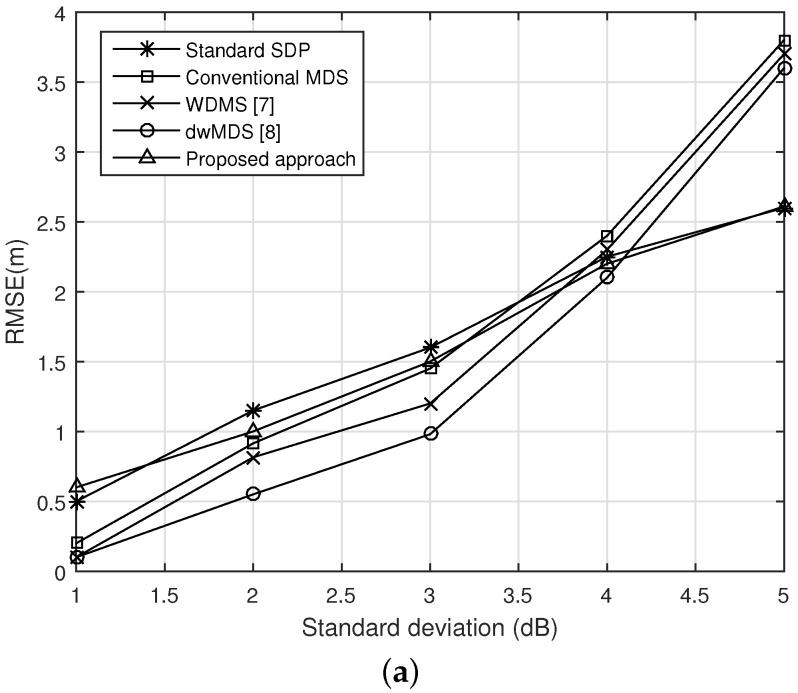

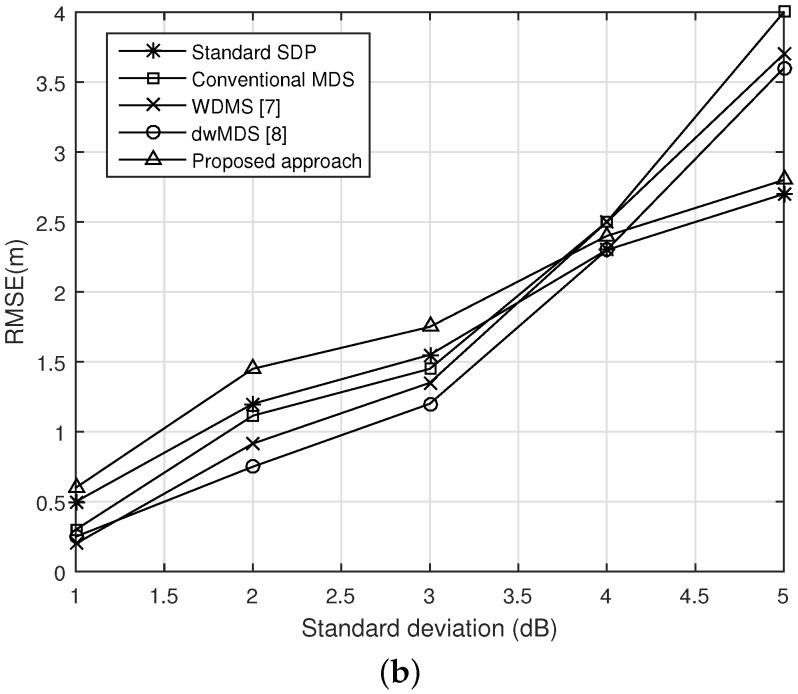
Comparison of root mean square error (RMSE) performance of our scheme and several methods *versus* *σ*. (**a**) A good precision for P; (**b**) A bad precision for P.

For the purpose of comparison, the RMSEs of our scheme and several schemes *versus* *σ* are shown in Figure 4. Each result is collected after 1000 independent runs. After obtaining the distance information in Equation (12), we get the standard SDP estimator (SDP is used in the context of linear matrix inequalities and can be efficiently solved by interior point methods, which has the complexity of $O(N^{4.5}\ln\left(1/\epsilon \right)$), here, *ϵ* is a given solution precision) by using the CVX package [18,19]. From the figure, we observe that our solution achieve the solution that better than the conventional MDS, WMDS, and dwMDS methods when the noise is large, and quite comparable estimation accuracy with the standard SDP for a small value of *σ*, since our solution depends on how good initial points are. It can be improved if we can choose a good parameter to set up. Figure 4b give us such an example. However, note that in this particular simulation with MATLAB implementation, the proposed method is faster than the others in running time.

The results indicate that our scheme is more suitable for the case of limited anchor node information. This is a good property for some scenarios where anchor node positions are not available. Thus, the obtained solution can be provided as a good initial point for other fine localization algorithms.

## 5. Conclusions

In contrast to many existing algorithms developed for the Euclidean distance matrix completion problem or its relaxation, we cast the problem under unconstrained formulation in terms of the location vectors directly and propose a new scheme using a modified Newton’s method instead. We manage the complexity by organizing the gradient and Hessian matrices. The theory is simple, but the results are effective and robust for localization in WSNs for intelligent IoT.
